# Advanced Hybrid Closed Loop Systems in Pregnancy: A Retrospective Study of Women With Type 1 Diabetes

**DOI:** 10.1002/edm2.70195

**Published:** 2026-04-09

**Authors:** Dimitra Stathi, Katharine Hunt, Helen Rogers, Konstantinos S. Kechagias, Anna Brackenridge

**Affiliations:** ^1^ Department of Diabetes, King's College Hospital NHS Foundation Trust London UK; ^2^ Department of Endocrinology and Diabetes Guy's and St Thomas' NHS Trust London UK; ^3^ Department of Metabolism, Digestion and Reproduction, Faculty of Medicine Imperial College London UK

**Keywords:** hybrid closed loop systems, continuous glucose monitoring, HCLS, insulin pump, pregnancy

## Abstract

**Background:**

Hybrid closed loop systems (HCLS) lead to improved glycaemic outcomes without the increased burden of hypoglycemia. CamAPS and Mendtronic 780G are the only systems licensed for use in pregnancy in Europe; however, all the commercially available algorithms are currently used in clinical practice. The aim of this study is to share our experience of using HCLS during pregnancy.

**Methods:**

This is a retrospective study. We reviewed records at two teaching hospitals from January 2018–March 2023 and identified 42 women of whom 42.8% (*n* = 18) were established on HCLS prior to pregnancy and 57.2% (*n* = 24) started during pregnancy at a median of 12 weeks of gestation.

**Results:**

Time in pregnancy target range (TIRp) (3.5–7.8 mmol/L) increased from 57% (44.5%–65%) (median, IQR) at first visit to 73% (65%–82%) (*p* < 0.001) at 34 weeks of gestation with 66% achieving ≥ 70% at 34 weeks. Time below range (TBR) glucose did not change significantly. There was no significant difference in TIRp at 8 or 34 weeks between those started on HCLS before versus during pregnancy. TBR at first visit was lower in those established on HCLS pre‐pregnancy versus those started in pregnancy (p: 0.04) with no difference between groups at 34 weeks. There were no admissions for diabetic ketoacidosis (DKA) or severe hypoglycemia. Eleven infants (26.8%) had birthweight over 90th percentile and neonatal hypoglycemia was recorded in 9 cases (22%).

**Conclusions:**

In our cohort, HCLS in pregnancy was effective, with 66% achieving ≥ 70% TIRp, and appears to be safe with low TBR and no episodes of DKA.

## Graphical abstract

In a cohort of 42 women who used hybrid closed loop systems in pregnancy, time in the pregnancy target range (3.5–7.8 mmol/L) increased from 57% (44.5%–65%) at first visit to 73% (65%–82%) at 34 weeks (*p* < 0.001). Frequency of severe hypoglycaemia was low and there were no episodes of diabetic ketoacidosis.

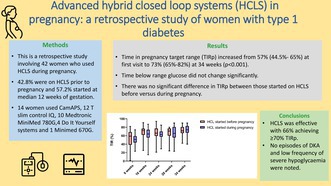

## 1 Introduction

Despite recent advances in diabetes care and the positive outcomes of continuous glucose monitoring (CGM), achieving the recommended glycaemic targets (3.5–7.8 mmol/L) during pregnancy remains challenging [1]. Pregnancy is characterised by increased glycaemic variability in early stages, altered insulin absorption and gradually rising insulin resistance and consequently increasing requirements.

Hyperglycemia is associated with a higher incidence of maternal and foetal complications including macrosomia, congenital abnormalities, prematurity, foetal loss and preeclampsia [2, 3]; therefore, optimal glycaemic management is crucial to reduce the risk of adverse outcomes. However, target HbA1c levels in early and late pregnancy are achieved in only 40% of the T1DM pregnancies in the UK [4]. There is also an increased frequency of preterm and large for gestational age (LGA) infants at 37% and 40% respectively in pregnancies complicated with T1DM.

Outside pregnancy, advanced hybrid closed loop systems (HCLS) have proven to be effective in improving glycaemic parameters and thus leading to tighter control without the increased burden of hypoglycaemia and have received positive feedback by users regarding their impact on quality of life and psychosocial benefits [5].

There are currently two commercially available HCLS algorithms approved for use in pregnancy (CamAPS FX, CamDiab Limited, UK; MiniMed 780G, Medtronic plc, Ireland/Global) in Europe. The CONCEPTT trial had previously demonstrated that CGM positively impacts neonatal outcomes and hyperglycemia [6, 7]. The AiDAPT randomised controlled trial (automated insulin delivery among pregnant women with type 1 diabetes) included 124 participants and showed better mean TIRp glucose in the CamAPS group compared to the standard‐care group (68.2% versus 55.6%, *p* < 0.001) with higher overnight TIRp glucose and less time in the hypoglycemic range [8].

The open‐label CRISTAL trial showed no difference in TIRp between Minimed 780G HCLS and standard insulin therapy; however, it reduced TBR and was not associated with any safety concerns [9]. A retrospective case series of eight women on T:slim, of whom 6 were on control IQ and 2 on basal IQ, showed variable success in achieving glycemic targets, with average TIRp at 67.9%, lower TBR, but higher mean glucose in those using control IQ [10]. A multicenter observational study in Spain that included 137 pregnant women with type 1 diabetes on CamAPS, MiniMed 780G or Control‐IQ showed that CamAPS FX and Control‐IQ users had better glycemic parameters and lower frequency of macrosomia compared with those using MiniMed 780G [11].

Existing literature and our own experience indicate that unlicensed, commercially available HCL systems are also used in pregnancy [12]: some are established on the HCLS prior to pregnancy; some are already on a stand‐alone pump, start‐CGM in pregnancy and are therefore able to activate HCLS; and some may choose to start a particular system in pregnancy.

The aim of this retrospective study is to share our experience of HCLS during pregnancy in women with type 1 diabetes.

## 2 Methods

### 2.1 Data Extraction

This is a retrospective study. We identified pregnant women with type 1 diabetes who delivered or experienced miscarriage between 1st January 2018 and 31 March 2023, used HCLS during pregnancy and were under the care of Guy's and St Thomas' NHS Trust or King's College Hospital NHS Foundation Trust. Both hospitals maintained databases of women with type 1 diabetes who were followed up in the diabetes clinic during the specified period.

We retrospectively reviewed electronic healthcare records (including BadgerNet Maternity, electronic patient records, E‐noting) to obtain demographic, clinical and admission data. We obtained pump and CGM data from Libreview, Dexcom Clarity, Carelink, Diasend, Glooko and Nightscout. CGM reports were adjusted for the recommended targets for pregnancy: 3.5–7.8 mmol/L [1]. Overnight CGM data was not analysed separately as we did not extract time‐segmented glucose data retrospectively. All information was pseudoanonymized as per General Data Protection Regulation (GDPR).

For baseline data (at first visit in pregnancy), we obtained: age, ethnicity, IMD score (derived from postcode using https://www.fscbiodiversity.uk/imd/), duration of diabetes, smoking status, BMI, comorbidities including hypertension, presence of retinopathy, history of renal disease, diabetes treatment before pregnancy, start dates for HCLS and system used, type of insulin used, total daily insulin dose (TDD), episodes of diabetic ketoacidosis (DKA) and severe hypoglycaemia within the last year.

We obtained data at five timepoints during pregnancy: first visit (between 6 and 8 weeks) and at the time of routine face‐to‐face antenatal diabetes clinic appointments, which were scheduled at approximately 16, 24, 28 and 32 weeks of gestation. CGM and pump data were examined for time in pregnancy target range (TIRp), time above range (TARp) and below range (TBRp), average glucose and TDD (basal and bolus) adjusting the dates to reflect the above stages of gestation and for the duration of 1 week. Clinical records were examined for laboratory HbA1c, Gold scores, severe hypoglycaemic episodes defined as hypoglycaemia that required third party assistance for correction and management, and DKA events. For those who started HCL during pregnancy, start date and system were recorded.

### 2.2 Maternal and Neonatal Outcomes

For maternal outcomes, we recorded mode of birth and use of pump therapy/HCLS during birth, preeclampsia and progression of retinopathy during pregnancy. We also looked at the CGM data for the first 2 weeks following birth and recorded TIR and TBR defining target glucose range as 3.9–10 mmol/L.

For neonatal outcomes, we recorded pregnancy loss, preterm birth defined as < 37 weeks and early preterm birth defined as < 34 weeks, birth weight and birthweight percentiles adjusted for weeks of gestation, birth injury, shoulder dystocia, neonatal hypoglycaemia defined as glucose < 2.6 mmol/L, hyperbilirubinemia and hypothermia.

This study was designed as a descriptive retrospective study. Participants used several commercially available HCL systems; however, device allocation was not randomised, sample sizes for individual systems were small, and the study was not powered to compare glycaemic outcomes between systems. Therefore, system‐specific analyses and comparison were not undertaken.

### 2.3 Statistical Analysis

Variables were tested for normality by Shapiro–Wilk test and Q–Q plots. Mean and standard deviation were calculated for the normally distributed variables and median and lower and upper quartile for the non‐normally distributed variables. To compare variables between the two groups, Student's *t*‐test was performed for normally distributed variables and Kruskal–Wallis for non‐normally distributed variables. Two‐way ANOVA was used to examine differences at different timepoints in the whole cohort. CGM and pump data were examined for artefacts. Days with < 70% usable data were excluded from glycaemic‐metric calculation. All preprocessing procedures were applied consistently across participants prior to analysis. All statistical analysis was done with IBM SPSS Statistics version 27.0. A *p* value < 0.05 was considered statistically significant.

## 3 Results

We identified 42 pregnant women with type 1 diabetes who used HCLS during their pregnancy and who delivered between January 2018 and March 2023. Eighteen women (43%) were already on HCLS prior to pregnancy, while 24 women (57%) commenced during pregnancy, of whom 10 (42%) were started on HCLS before 12 weeks of gestation and 13 (54%) between 12 + 1 and 22 + 4 weeks of gestation. One woman was started on HCL late in pregnancy at 31 weeks of gestation and was excluded from the analysis. Data are reported for 41 women. There were no women with a second pregnancy in the above period.

Demographics, baseline characteristics and co‐morbidities are provided in Table 1. Mean age at first visit in the antenatal diabetes clinic was 34.2 ± 4.2 years (mean ± SD) and mean duration of diabetes 19.8 ± 7 years. Most patients were of white ethnicity (85.3%, *n* = 35). We categorised individuals into ‘low’ (deciles 7–10), ‘medium’ (4–6) and ‘high’ (1–3) groups based on their Index of Multiple Deprivation (IMD) deciles, with low IMD indicating the least deprived group and high IMD showing high deprivation. The majority was in the low IMD group (*n* = 19, 45.2%) followed by 10 women (23.8%) and 13 women (31%) in the medium and high groups, respectively. At the first visit, BMI was 25 (23.1–27.7) kg/m^2^ (median, IQR). No one was an active smoker. Hypertension was recorded in 12.1% (*n* = 5), retinopathy in 56.1% (*n* = 23; R1: *n* = 17, R2: *n* = 2, R3: *n* = 4) and chronic kidney disease in 4.8% (*n* = 2).

**TABLE 1:** Baseline characteristics.

	All patients (*n* = 41)	HCLS prior to pregnancy (*n* = 18)	HCLS started in pregnancy (*n* = 23)
Age (years) (Mean ± SD)	34.2 (±4.2)	34.1 (±4.7)	34.3 (±4)
Ethnicity (*n*, %)	Caucasian: 35 (85.3%) African Caribbean: 4 (9.7%) Asian: 1 (2.5%) Other: 1 (2.5%)	Caucasian: 17 (94.5%) Other: 1 (5.5%)	Caucasian: 18 (78.2%) African Caribbean: 4 (17.3%) Asian: 1 (4.5%)
Mean duration of diabetes (years) (Mean ± SD)	19.8 (±7)	19.8 (±6.4)	19.8 (±7.6)
BMI (kg/m^2^) (median–IQR)	25 (23.1–27.7)	25 (24.3–27.9)	25.7 (23–28)
Retinopathy^a^	R0: 18 (43.9%) R1: 17 (41.5%) R2: 2 (4.9%) R3: 4 (9.7%)	R0: 6 (33.3%) R1: 9 (50%) R2: 1 (5.5%) R3: 2 (11.2%)	R0: 12 (52.2%) R1:8 (34.8%) R2:1 (4.4%) R3:2 (8.7%)
Hypertension (*n*, %)	5 (12.1%)	3 (16.6%)	2 (8.6%)
CKD (*n*, %)	2 (4.8%)	Normal renal function: 16 (88.8%) Stage 1: 1 (5.6%) Stage 3: 1 (5.6%)	0% (*n* = 0)
Smoking (*n*, %)	Never smoked: 38 (92.6%) Ex‐smokers: 3 (7.3%)	Never smoked: 16 (88.8%) Ex‐smoker: 2 (11.2%)	Never smoked: 22 (95.6%) Ex‐smoker: 1 (4.4%)

^a^ Two patients with R1 also had maculopathy.

Of the 18 individuals using HCLS prior to pregnancy, 6 individuals (33.3%) were using Tandem T slim control IQ, 5 (27.7%) Medtronic MiniMed 780G, 4 (22.3%) were using the Do‐it‐yourself algorithms (DIY), 2 (11.2%) were on CamAPS with Ypsomed pump and Dexcom CGM and 1 (5.5%) was on Medtronic MiniMed 670G (Table 2). The majority used Novorapid (*n* = 16; 88.8%) followed by Humalog (*n* = 1; 5.6%) and Fiasp (*n* = 1; 5.6%). Of the women who were started on HCLS during pregnancy, 10 (43%) were previously on basal bolus insulin regimen, while 13 (57%) were on CSII. Among them, 12 women (52%) started CamAPS during pregnancy, 6 Control IQ (26%) and 5 Medtronic 780G (22%). Novorapid was predominantly used (*n* = 18; 78.3%) followed by Humalog (*n* = 5; 21.7%).

**TABLE 2:** Comparison of glycaemic parameters between previous and new HCLS users.

	HCLS prior to pregnancy (*n* = 18)	HCLS started in pregnancy (*n* = 23)	*p*
First visit (6–8 weeks)			
Median TIRp (%)	59.5 (47–70)	49 (43.5–58)	0.1
Median TBRp (%)	1 (1–2)	2 (0–9)	0.04
Median glucose (mmol/L)	7.4 (7–8.7)	8.1 (7.3–8.7)	0.3
Severe hypos	0	1	—
DKAs	0	0	—
Median basal TDD (units)	18 (14.3–26.9)	23 (17.7–33)	0.2
Median bolus TDD (units)	21 (14–36)	18.5 (14.5–29.5)	0.4
HbA1c 1st trimester (mmol/mol)	47 (45.4–54.1)	53 (45.4–61.7)	0.1
34 weeks			
Median TIRp (%)	71.5 (67–80)	74 (66–82.5)	0.9
Median TBRp (%)	1 (0–1)	2 (1–4)	0.04
Median glucose (mmol/L)	6.9 (6.4–7.1)	6.7 (6–7)	0.7
DKAs	0	0	—
Severe hypos	0	0	—
Median basal TDD (units)	22 (16–26.3)	27 units (19.7–41.1)	0.09
Median bolus TDD (units)	30.7 (25.5–36.5)	42 units (25.3–56)	0.2
HbA1c 3rd trimester (mmol/mol)	41 (38.8–51.9)	42.1 (36.6–50.3)	0.6

Across the whole cohort, the most commonly used Hybrid Closed Loop system during pregnancy was CamAPS (34.1%, *n* = 14) of whom 12 were taking part in the AiDAPT study [5], followed by Tandem T slim control IQ (29.2%, *n* = 12) and Medtronic MiniMed 780G (24.3%, *n* = 10). One individual was on Medtronic Minimed 670G, while 4 continued using Do It Yourself (DIY) systems (Table 2).

Median TIR glucose and IQR before pregnancy was 76% (66.2%–82.5%) in the group of HCLS users and 66.5% (47%–73%) in the non‐HCLS users that were on either basal bolus or standalone pump therapy prior to pregnancy (*p*: 0.03). The latter also had a higher time below range [3% (2%–8%)] compared to HCLS users [1.5% (1%–3%)] (*p*: 0.02). HCLS users had a median HbA1c of 6.8% (6.4%–7.3%) [50.8 (46.4–56.3) mmol/mol] and non‐HCLS users 7.05% (6.45%–8.05%) [53.6 (47–64.5) mmol/mol] prior to gestation (*p*: 0.1). There were no severe hypoglycaemic episodes or admissions for DKA reported in either group within the last 6 months (Table 3).

**TABLE 3:** Glycaemic parameters of the cohort during different stages of pregnancy.

All patients (*n* = 41)	First visit	16 weeks	24 weeks	28 weeks	34 weeks
TIRp (%) (median, IQR)	57 (44.5–65)	67.5 (59.5–75)	65 (59–73)	66 (56.5–77)	73 (65–82)
TAR (%) (median, IQR)	40 (30, 52.5)	29 (22, 37.5)	33 (29, 39)	30 (20.5, 41)	26 (15, 32)
TBR (%) (median, IQR)	2 (1–8.5)	2 (1–4)	2 (1–3)	1 (0–3)	1 (1–3)
Average glucose (mmol/L) (median, IQR)	7.6 (7.2, 8.7)	6.9 (6.5–7.1)	7.2 (6.9–7.7)	7.1 (6.5, 7.9)	6.8 (6.4–7.2)
Basal TDD (units) (median, IQR)	22 (16–30)	17 (13–25)	22 (16–31.3)	24 (18.7–32.3)	26.3 (17.1–35.6)
Bolus TDD (units) (median, IQR)	19 (12.5–34.5)	21 (15.5–34)	27 (20–36)	29.9 (25–49)	34.4 (26–45.1)
DKA since preceding visit (*n*)	0	0	0	0	0
Severe hypoglycaemic episode (*n*)	1	1	0	0	0

Table 2 shows glycaemic data for those who started HCLS prior to pregnancy and those who started HCLS in pregnancy. Those who started HCLS in pregnancy started after the first visit and before the 24‐week visit. Table 3 shows glycaemic parameters for the whole cohort through pregnancy. TIRp increased from 8 to 34 weeks of gestation. TIRp glucose levels increased from 57% (44.5–65) at first visit to 67.5% (59.5%–75%) at 16 weeks (*p* < 0.0001), plateaued between 16 and 28 weeks and increased again from 66% (56.5%–77%) at 28 weeks to 73% (65%–82%) (*p*: 0.01) at 34 weeks. TBRp decreased from 2% (1%–8.5%) at first visit to 1% (1%–3%) at 34 weeks (*p*: 0.002).

Average glucose decreased from 7.6 (7.2–8.7) mmol/L at first visit to 6.8 (6.4–7.2 mmol/L) at 34 weeks. Basal total daily insulin dose (TDD) increased from 22 (16–30) units at first visit to 26.3 (17.1–35.6) units at 34 weeks. Bolus TDD increased from 19 (12.5–34.5) units at first visit to 34.4 (26–45.1) units at 34 weeks (Table 2). Two hypoglycaemic episodes that required third‐party assistance were reported, both in the group who started hybrid closed loop in pregnancy, one at first visit prior to HCLS initiation and one at 16 weeks within the first week of starting closed loop therapy. No admissions for diabetic ketoacidosis (DKA) were recorded.

At first visit, comparing the group who started HCLS in pregnancy (were not using HCLS at first visit) with those who were using HCLS prior to pregnancy: TIRp was 49% (43.5%–58%) versus 59.5% (47%–70%) respectively (*p* = 0.1); TBRp was higher [2% (0%–9%) vs. 1% (1%–2%)] (*p* = 0.04), HbA1c was 7% (6.3%–7.8%) [53 (45.4–61.7) mmol/mol] versus 6.5% (6.3%–7.1%) [47.5 (45.4–54.1) mmol/mol] (*p* = 0.1); at 34 weeks, TIRp was similar at 74% (66%–82.5%) versus 71.5% (67%–80%) (*p* = 0.9); TBRp remained higher at 2% (1%–4%) and 1% (0%–1%) (*p* = 0.04), total daily basal insulin was higher at 27 (19.7–41.1) units versus 22 (16–26.3) units (*p* = 0.09) and HbA1c was similar at 6% (5.5%–6.7%) [42.1 (36.6–50.3) mmol/mol] versus 5.9% (5.7%–6.9%) [41 (38.8–51.9) mmol/mol] (*p* = 0.6).

**FIGURE 1:**
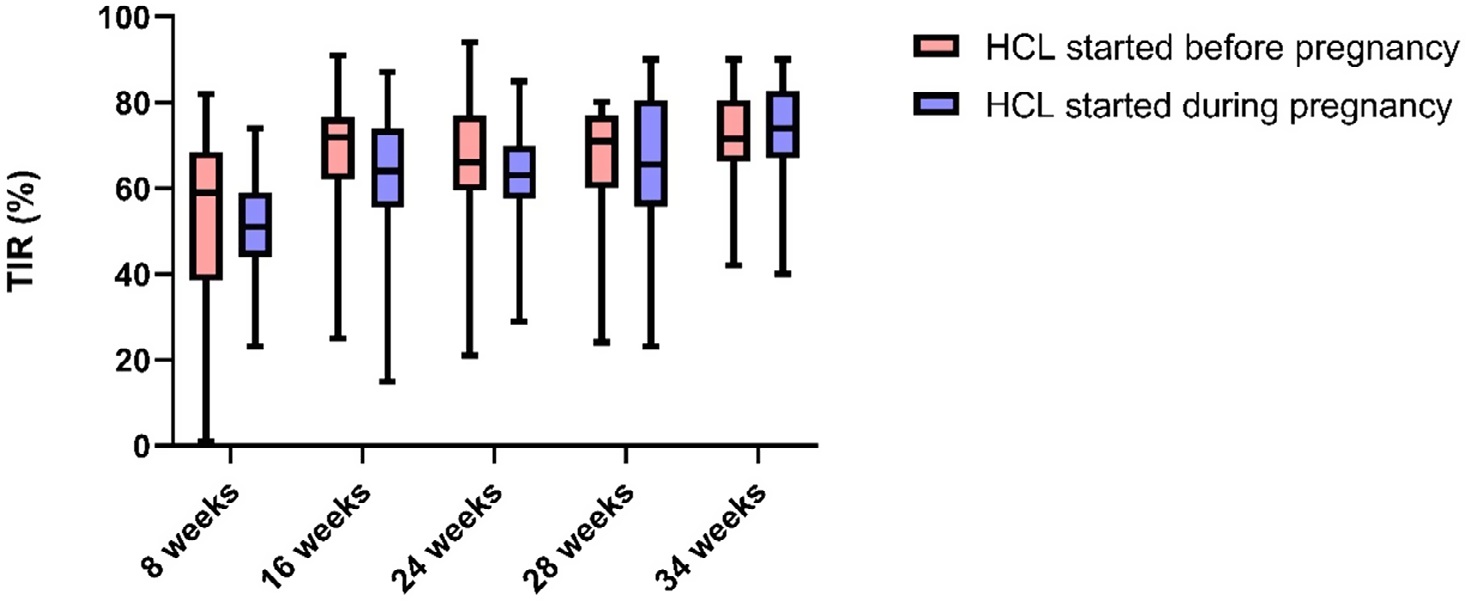
Comparison of TIRp during pregnancy in those started on HCL before pregnancy and those who started during pregnancy.

Data on TIRp glucose during pregnancy in the two groups are presented in Figure 1. The wide variability observed in the boxplots, especially at earlier gestational timepoints, reflects heterogeneity in glycaemic management across participants. This is partly explained by differences in baseline glycaemic management, timing of HCLS initiation and individual adaptation to the system. As pregnancy progressed, interquartile ranges narrowed, suggesting more consistent system use and improved metabolic control across the cohort.

Three women developed pre‐eclampsia (7.3%). Three women (7.3%) developed or had progression of retinopathy/maculopathy; prior to pregnancy they had background retinopathy and two of them had suboptimal glycaemic management with HbA1c at 8.1% (65 mmol/mol) and 8.4% (68.3 mmol/mol) respectively. Caesarean section was performed in 58.6% of the cases (*n* = 24). Half of the cohort (51.2%, *n* = 21) remained on HCLS during birth, while the remaining patients were switched to intravenous (IV) insulin (variable rate) with IV glucose. There was no twin pregnancy. There was no foetal loss after 12 weeks or injury during labour. Pre‐term delivery (< 37 weeks) was noted in 13 cases (31.7%) and early pre‐term (< 32 weeks) in 4 cases (9.7%). Mean birth weight in full term infants was 3.344 (±84.9) gr with 2 infants weighing more than 4 kg. Eleven infants (26.8%) had birthweight over the 90th percentile. Neonatal hypoglycaemia was noted in 22% of the cases (*n* = 9) and jaundice in 29.2% (*n* = 12) (Table S1).

Our usual practice is for women to continue using hybrid closed loop post birth, with adjustments in targets or using the settings recommended for exercise depending on the system to make the algorithm less aggressive. CGM data for the first 2 weeks following birth were available for 34 (of 41). TIR glucose (range 3.9–10 mmol/L) remained optimal at 82.5% (72%–88%) with TBR glucose at 2% (1%–5%).

## 4 Discussion

We present a retrospective study of 41 women with type 1 diabetes who used a range of currently available hybrid closed loop systems during pregnancy and the early postnatal period. Our data add to the growing evidence that technology can be safely implemented in antenatal care and support improved glycaemic management throughout gestation.

Women in our cohort used 5 different systems including CamAPS, Tandem Control IQ, MiniMed 780G, DIY and Minimed 670G. Importantly, 28.5% of the women were randomised to CamAPS HCL within the AiDAPT trial. In our centres, access to CGM and pump therapy was facilitated by national guidance and available funding. All patients on intensive insulin therapy with an HbA1c of ≥ 6.5% were informed about the AiDAPT trial, which was offered at the time in both hospitals. Problematic hypoglycaemia, impaired hypoglycaemia awareness and suboptimal or erratic glucose levels on multiple daily injections despite intensive follow‐up were other driving factors for starting HCLS before or during pregnancy.

Across all systems, HCLS were generally safe and effective. There were no episodes of DKA. There was one episode of severe hypoglycaemia when using hybrid closed loop. This occurred within the first week of starting hybrid closed loop therapy in pregnancy (discussed further below). This emphasises the importance of close monitoring, individualised structured education and frequent follow‐up provided within a multi‐disciplinary clinic framework. Patients should be encouraged to take prompt action when experiencing unexplained hyperglycaemia or illness. Across all systems, optimisation of settings, including review of insulin carbohydrate ratios and sensitivity factors, meal bolus timing and use of tight glucose targets, when available, were crucial. However, since not all systems are specifically designed to achieve the stricter glycaemic targets, in some cases additional correction boluses or fake carbohydrates were required to maintain optimal glucose control.

TBR remained low throughout pregnancy, while TIRp increased from 57% in early pregnancy to around 68% by 16 weeks of gestation, remained static until 28 weeks of gestation then improved further to 73% by 34 weeks of gestation. Overall, 66% of women achieved TIRp glucose ≥ 70% at 34 weeks. Our results are comparable to those of the AiDAPT trial, suggesting that increasing HCLS familiarity likely contributes to improved glycaemic outcomes as pregnancy progresses.

Initiation of HCLS in pregnancy appeared to be generally effective. The group who started HCLS before pregnancy and those who started HCLS in pregnancy (up to 22 + 4 weeks of gestation) were broadly similar in terms of demographics. At first visit, those who were already on HCLS had lower TBRp compared to those who went on to start HCLS in pregnancy, and other glycaemic parameters (TIRp, average glucose, HbA1c) also appear to favour starting HCLS prior to pregnancy, although no statistically significant differences were found. These findings are consistent with previous data regarding HCLS [8].

Variability in glycaemic parameters, especially in early pregnancy, reflects the heterogeneity of a real‐world cohort: women differed in baseline glucose levels, timing of HCLS initiation and the system used, and some were already experienced pump users prior to pregnancy. Furthermore, the first trimester is characterised by fluctuating insulin requirements which can also contribute to glucose variability.

Larger studies, ideally RCTs, in the preconception period could provide further information on the effectiveness of these systems. Given optimal glycaemic management (HbA1c < 6.5%) reduces the risk of congenital abnormalities, perinatal death, preterm birth, large for gestational age (LGA) birthweight and neonatal care admissions, early use of HCLS during preconception planning is expected to be beneficial [13]. However, by 34 weeks of gestation, TIRp, median glucose and HbA1c were similar in the two groups with the exception of TBRp, which continued to be lower in those who started HCLS prior to pregnancy.

Obstetric and neonatal outcomes (Table S1) in our cohort were broadly comparable to those reported in the national audit and interventional studies. Caesarean section was the most common mode of delivery. There was no neonatal death or injury. Rates of preterm birth (41.4%) were similar to existing literature, although the proportion of LGA infants (26.8%) and neonatal hypoglycaemia (22%) were lower than reported in other closed‐loop trials such as AiDAPT and Cristal [3, 8]. Evidence suggests that foetal hyperinsulinemia can persist despite maternal normoglycaemia, which could explain the above findings [8, 9, 14, 15].

Continuation of HCLS post‐birth appeared safe, with high TIR and low TBR, despite rapidly falling insulin requirements in the early postnatal period (2 weeks post‐birth). With adaptive HCLS, where the algorithms use information about insulin requirements in the preceding days (all the HCLs included here except Tandem Control IQ), there is concern they may not learn quickly enough and therefore might increase the risk of hypoglycaemia. Our usual practice is for women to continue using HCLS post birth, with adjustments to make the algorithm less aggressive by modifying the targets and/or using the settings usually recommended for exercise depending on the system. Of course, as with any pump system, the insulin carb ratio (ICR), insulin sensitivity factor (ISF), bolus calculator target and programmed basal must all be changed to post‐pregnant settings (whether they are used by the HCLS algorithm to adjust insulin delivery while in HCLS or required as back‐up in case out of HCLS). In our cohort of 34 women who continued to use HCLS in the 2 weeks post‐birth, there was low TBR at 2% and high TIR at 83%. We are not able to comment on whether there were any severe hypoglycaemic episodes.

One of the strengths of our study is the number of patients included, compared to most of the previously published case reports and studies. Additionally, we share our experience on all commercially available HCLS with data recorded before, throughout pregnancy, and during the first 2 weeks post‐delivery. We were also able to investigate the differences in glycaemic management in women who were using HCLS prior to pregnancy and those started on closed loop during gestation.

However, this is a retrospective study and not a randomised controlled trial; hence we are not able to make comparisons between the different HCLS, and there was no comparator group such as standalone pump or multiple insulin injection users. Moreover, we did not collect data on well‐being, and we were not able to collect CGM data during labour. In our analysis, we did not control for confounding variables such as age and ethnicity. We also acknowledge that 28.5% of our cohort participated in the AiDAPT trial.

## 5 Conclusions

We observed improved time in range and time below range glucose with the use of all commercially available HCLS in pregnancy with no episodes of DKA or high frequency of severe hypoglycaemia and overall positive neonatal and maternal outcomes.

While preliminary observations suggest potential benefits of HCLS in pregnancy, clinical trials are needed to rigorously evaluate and compare the efficacy and safety of the available systems, and to better understand their potential risks and advantages in this specific population.

## Author Contributions

D.S.: conceptualization, data collection, formal analysis, writing – original draft, editing. K.H.: conceptualization, supervision, methodology, writing, editing and review. H.R.: data collection, editing and review. K.S.K.: formal analysis, editing and review. A.B.: conceptualization, supervision, methodology, writing, editing and review.

## Funding

The authors have nothing to report.

## Conflicts of Interest

The authors declare no conflicts of interest.

## Supporting information


**Table S1:** Maternal and infant outcomes.

## Data Availability

The data that support the findings of this study are available from the corresponding author upon reasonable request.
