# Supplementary material for: Advanced Hybrid Closed Loop Systems in Pregnancy: A Retrospective Study of Women With Type 1 Diabetes
Source: Endocrinol Diabetes Metab. 2026 Apr 9;9(3):e70195. doi: 10.1002/edm2.70195 (PMC13065487; doi:10.1002/edm2.70195)
Supplement: Supplementary file 1 — Table S1: Maternal and infant outcomes. [file EDM2-9-e70195-s001.docx]

**Table S1: Maternal and infant outcomes**

| **Delivery /Post-delivery data** | |
| --- | --- |
| Type of delivery | C-section: 24 (58.6%)  Vaginal birth: 15 (36.9%)  Not available (NA): 2 (4.5%) |
| Progression of retinopathy | 7.3% (n=3) |
| Preeclampsia | 7.3% (n=3) |
| Use of pump during delivery | Yes – 22 (53.9%)  No- 18 (43.9%)  NA- 1 (2.5%) |
| Use of HCLS during delivery: | Yes – 21 (51.2%)  No – 18 (44%)  NA – 2 (4.8%) |
| TIR 2 weeks after delivery (n=34) | 82.5 (72- 88) |
| TBR 2 weeks after delivery (n=34) | 2 (1-5) |
| **Infant outcomes** | |
| Mean birth weight (gr) | 3.344 (±84.9) |
| LGA | 26.8% (n=11) |
| Pregnancy loss | 0% (n=0) |
| Pre-term | 31.7% (n=13) |
| Early pre-term | 9.7% (n=4) |
| LGA | 26.8% (n=11) |
| Neonatal injury | 0% (n=0) |
| Shoulder dystocia | 2.4% (n=1) |
| Neonatal hypoglycaemia | 21.9% (n=9) |
| Jaundice | 29.2% (n=12) |
| Hypothermia | 4.8% (n=2) |
